# Evaluation of a health-related intervention to reduce overweight, obesity and increase employment in France and the United Kingdom: a mixed-methods realist evaluation protocol

**DOI:** 10.1186/s12889-021-10523-3

**Published:** 2021-03-24

**Authors:** Sophia D. Amenyah, Jane Murphy, Lee-Ann Fenge

**Affiliations:** Faculty of Health and Social Sciences, 5th Floor, Bournemouth Gateway Building, Bournemouth University, St Paul’s Lane, Bournemouth, BH8 8GP UK

**Keywords:** Obesity, Overweight, Unemployment, Realist evaluation, Healthy lifestyle, Employment, Quality of life

## Abstract

**Background:**

Obesity, overweight and unemployment are interlinked, with debilitating effects on mortality, health, wellbeing and quality of life. Existing interventions to reduce overweight, obesity and unemployment have addressed these challenges independent of each other with limited success. The Adding to Social capital and individual Potential In disadvantaged REgions (ASPIRE) project will develop an innovative model using a combination of skills training and health and wellbeing interventions to improve health, wellbeing, quality of life and reduce overweight, obesity and unemployment in England and France. The aim of this paper is to outline the protocol for evaluating the ASPIRE project to examine the effectiveness of the intervention and clarify the mechanisms and contextual factors which interact to achieve outcomes.

**Methods:**

A mixed-method realist evaluation using a single-group before-and-after design will be used. The evaluation will consist of development of an initial programme theory, theory validation and refinement using quantitative and qualitative data to understand the causal mechanisms, contexts of implementation and their interactions that result in outcomes observed in ASPIRE. Primary outcomes that will be assessed are change in body weight and body mass index, reemployment and a rise on the ASPIRE participation ladder. The ASPIRE participation ladders consists of a series of 5 steps to engage participants in the project. The first step on the ladder is joining an ASPIRE hub with paid employment as the final step on the ladder. Secondary outcomes will be physical activity, diet quality, self-efficacy and health-related quality of life. Both quantitative and qualitative approaches are appropriate in this study because the use of validated questionnaires and objective measures will demonstrate how much the intervention addressed outcomes related to weight loss and reemployment and the qualitative data (photovoice) will provide insights into the contexts and experiences that are unique to participants in the project.

**Discussion:**

The results from this evaluation will provide an understanding of how a model of health-related interventions which improve health, wellbeing and maintenance of a healthy lifestyle could reduce overweight, obesity and unemployment. The findings will enable the adaptation of this model for effective implementation in different contexts and circumstances.

**Trial registration:**

ISRCTN registry: Study ID: ISRCTN17609001, 24th February 2021 (Retrospectively registered).

**Supplementary Information:**

The online version contains supplementary material available at 10.1186/s12889-021-10523-3.

## Background

Obesity is a leading risk factor for the global burden of diseases [1] and a major contributor to all-cause mortality, morbidity and decline in both quality of life and life expectancy [2–4]. Of a greater concern is recent data showing that individuals with obesity are at a greater risk of morbidity and mortality from the coronavirus disease 2019 (COVID-19) [5–7]. Data from the Health Survey for England (HSE) indicates an overweight and obese prevalence of 66.9% in adult men and 59.7% in adult women [8]. In France, the prevalence of overweight (including obesity) in adults was 54% in men and 44% in women [9]. Apart from being a health risk factor, obesity is strongly associated with unemployment, social disadvantages and reduced socioeconomic productivity [10] and poor individuals in Europe are 10–20% more likely to be obese compared to individuals in high income brackets [11–13].

Consistent with global trends, obesity across the France Channel English (FCE) area (south and east coasts of England and the north coast of France) is a significant concern. In this region, high levels of obesity have been shown to coincide with high rates of unemployment. In the British household Panel Survey (BHPS), job loss was associated with weight gain of 1.56 kg/year and with significant decline in wellbeing and increased sleep deprivation [14]. Several causal pathways have been postulated to explain the link between unemployment or socioeconomic deprivation and high body mass index (BMI). Unemployment leads to more households experiencing a decrease in income, which calls for new strategies to cope with restrained household budgets and may lead to unhealthy diets. Individuals in lower income households are increasingly consuming diets which are able to satisfy caloric needs but are poor in micronutrient density, dietary variety and high in sugar and fat, leading to poorer health outcomes [15]. Data from the HSE survey shows that only 29% of adults consumed the recommended five portions of fruit and vegetables a day. Higher consumption of fruit and vegetables was also associated with higher income, and vice versa: 36% of all adults in the highest income quintile had consumed five or more portions of fruit and vegetables on the previous day compared with 23% of all adults in the lowest quintile [16]. Fresh, local, healthy food options are often more expensive and are more difficult to cook, therefore individuals cope by buying much cheaper food (highly processed meat products, high fat and sugar foods) or resort to ‘take-aways’ or ‘fast food’ which require no cooking [13, 17, 18].

Additionally, of those in the highest income quintile, 42% of men and 34% of women undertake at least five 30-min sessions of moderate or intensive physical activity (PA) per week, compared to 31 and 26%, respectively, in the lowest quintile [19]. Data from the French Health Study on Environment, Biomonitoring, Physical Activity and Nutrition (Esteban) showed that 53% of women and 71% of men achieved the World Health Organisation (WHO) recommendations on physical activity for health however, 90% of adults reported more than 3 h of sedentary activities per day and 42% of adults more than 7 h [20]. More recent data also showed that during the coronavirus pandemic, half of the population did not meet the recommendations for physical activity and one third reported a high level of sedentary lifestyle [20]. Insufficient physical activity was also more prevalent in people in lower professional categories, with no professional activity, women with fewer qualifications, time off work or partially unemployed [20].

In contrast obesity is considered a cause for lower income when obese people drift into lower-income jobs due to labour–market discrimination and public stigmatisation [21]. Research suggests that obese individuals are more likely to be perceived as lazy, unsuccessful, weak-willed and undisciplined resulting in negative discrimination due to body weight in the labour market, including higher job insecurity and lower chances of obtaining a job [22, 23]. In a longitudinal study using data from over 120,0000 adults across 21 European countries, obesity decreased employment chances and chronic conditions linked with high BMI negatively affecting employment likelihood and increased the intention to retire early [24]. Furthermore, psychological distress and subsequent emotional eating as a consequence of both obesity and unemployment provide a serial pathway linking unemployment to obesity. Maladaptive coping strategies, such as eating energy-dense foods to alleviate negative emotions and stress, coupled with stress-induced disturbances to metabolic signals promote weight gain and obesity over time [25]. The negative social, psychological, emotional, and behavioural consequences of obesity exacerbate psychological distress and maladaptive eating behaviours, can thus create a cyclic mechanism [25].

Although it has been well established that obesity and unemployment are strongly linked, existing services to tackle obesity and unemployment rarely work together to address the issue holistically. Additionally, there is a lack of interventions examining how the underlying causes of unemployment and obesity can be addressed using common strategies. The main public health interventions used to tackle obesity focus on information campaigns, advertising, labelling rules and regulation of nutritional claims [26]. While these types of interventions inform people about food characteristics, they are not able to successfully induce people to make healthier food choices. Interventions focused on improving the income of economically disadvantaged individuals with the additional effect of improving health are required. Further research is also needed on how individuals with obesity can best be supported to obtain and maintain employment.

The Adding to Social capital and individual Potential In disadvantaged REgions (ASPIRE) is an innovative project which seeks to create a new model for service delivery combining healthy weight and employability services to address unemployment and obesity in the FCE region. The ASPIRE project will target the obese, overweight and unemployed population across the FCE zone, using food production as a way to increase awareness and engagement, reduce weight, increase self-esteem, improve employability via new skills and work experience. ASPIRE will improve the quality and effectiveness of service delivery to socio-economically disadvantaged communities by co-ordinating healthy lifestyle opportunities with a pathway into employment. The health-based interventions co-created with ASPIRE partners will be adapted to socially and economically disadvantaged communities to reduce obesity and overweight and increase employability.

This paper is a protocol outlining the evaluation of the ASPIRE model. The aim will be to evaluate the ASPIRE model using Realist Evaluation (RE) methods to examine which ASPIRE interventions work, for whom, why and under what contexts.

## Methods

**Fig. 1:**
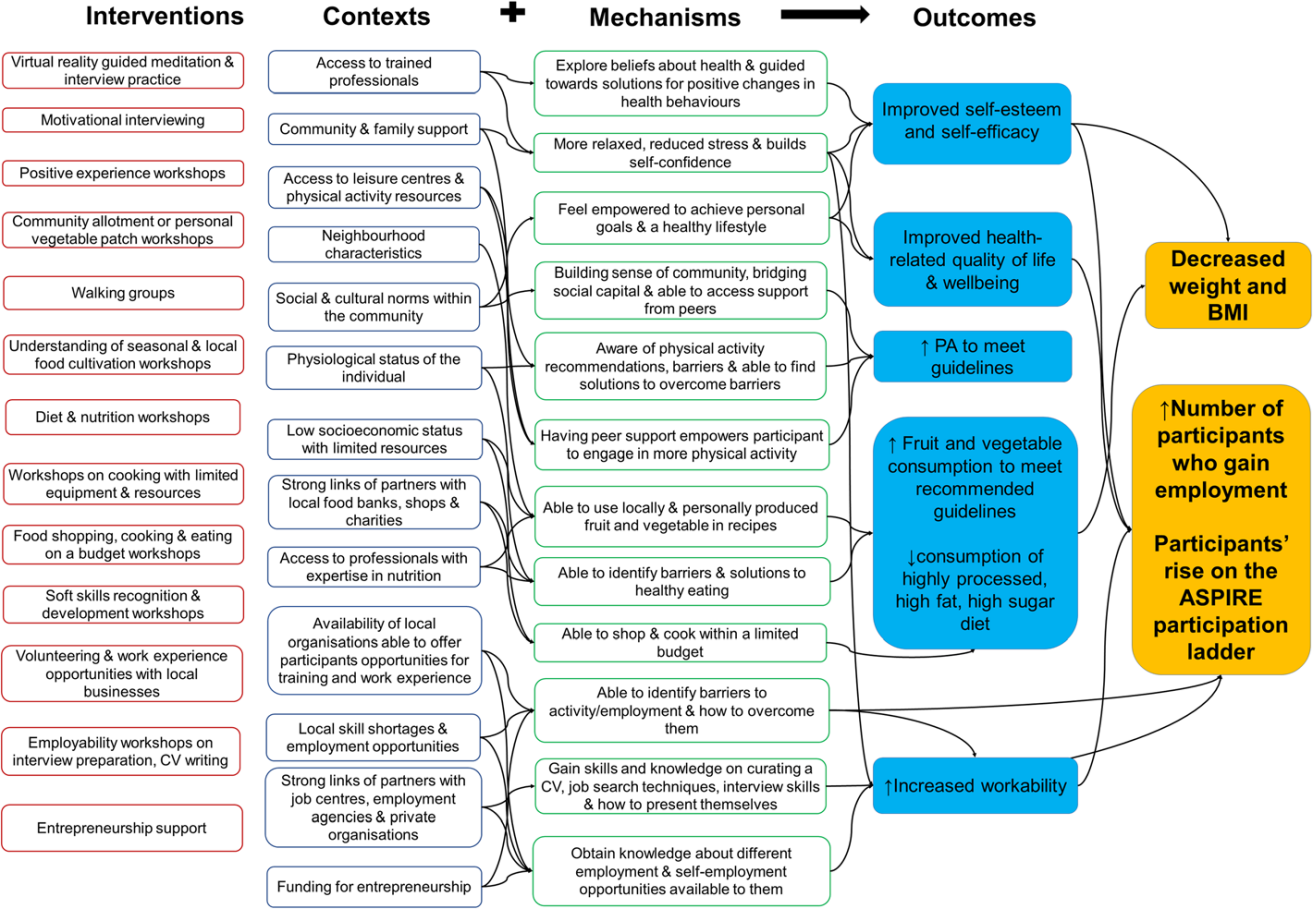
Conceptual framework illustrating the contexts-mechanism-outcome configuration underlying the ASPIRE model. **Abbreviations:** BMI, body mass index, CV, curriculum vitae, PA, physical activity

This evaluation is designed as a multidisciplinary and mixed-method process and will use RE methods to examine the effectiveness of the ASPIRE model. Realist evaluation uses key linked concepts (‘mechanism’, ‘context; and ‘outcomes’) for explaining and understanding programmes. This is known as the CMO configuration [27]. A CMO configuration is a proposition stating what it is about an intervention that works, for whom and in what circumstances. This approach will enable the development, validation and refinement of mid-range theories that account for how the *Context* in which ASPIRE intervention activities are implemented influence intervention *Mechanisms* (e.g. participants reasoning in uptake of interventions) to produce intended and unintended *Outcomes* (decrease in weight, BMI or unemployment). Realist evaluations assume that the success or failure of interventions are dependent on certain conditions, complex interactions of causal mechanisms and are heavily influenced by the way that different stakeholders respond to them [27]. Context refers to broad social or geographical features as well as factors affecting the implementation of programs (e.g. setting of intervention, adequate funding, the qualifications of staff) [27–29]. The context within which a project is implemented can influence the way in which, or the extent to which, a programme is implemented, who it targets and who it reaches. The mechanism is the underlying causal process which informs how and why an intervention works and for which participants [27]. Mechanisms can be intended and unintended, generating both positive and negative outcomes and are filtered through people, who have an ability to interpret and respond to them differently [27, 28, 30]. Therefore, evaluation of an intervention’s effectiveness should include how different people experience and respond to it and why. The RE method is particularly suited to evaluating new and complex interventions that seem to work but ‘for whom and how’ is not yet understood. While several of the interventions incorporated in the ASPIRE model have been used to independently reduce overweight or obesity and unemployment, there is limited evidence on how the combination of such interventions work holistically to reduce obesity and unemployment. The conceptual framework using CMO configuration to map the pathway from intervention to outcomes is illustrated in Fig. 1. This framework will be continuously validated and refined during data collection and analysis. The conduct and reporting of the evaluation will be guided by the Realist and Meta-Review Evidence Synthesis Evolving Standards (RAMESES II) reporting standards for realist evaluations [31].

### Initial Programme theory

The initial programme theory represents the underlying assumptions about how interventions implemented in ASPIRE are meant to work and what impacts they are expected to have. The development of the initial programme theory will consist of a critical realist synthesis of international literature to identify interventions used to reduce overweight, obesity and unemployment. Additionally, information on common approaches used in these interventions, the contexts and mechanisms that have contributed to the success or failure of the interventions will also be extracted to help refine the final programme theory. Gap and strengths, weaknesses, opportunities, threats (SWOT) analyses will also be conducted in collaboration with stakeholders to help identify existing resources within organisations which could enhance the implementation of the ASPIRE model. Furthermore, weaknesses and threats to the project will be identified and strategies will be implemented to mitigate the risks identified. An evaluation toolkit comprising of qualitative and quantitative data collection methods and tools have been identified and co-created with partners to be used with ASPIRE participants and providers to evaluate the efficacy of the ASPIRE project over its life course. The completed detailed programme theories developed will help to explore the relations between context, processes or mechanisms and outcomes (CMO configurations) to aid the refinement of the final programme theory.

### Final Programme theory

Theory refinement and validation will include adaptation and pilot testing of the qualitative and quantitative data collection tools, followed by subsequent data collection on outcomes to validate and refine programme theories. Different qualitative and quantitative methods will be used to understand and validate programme theories that link context, mechanisms and outcomes of the ASPIRE model. Subgroup analyses grouped by responders vs. non-responders, sex, site location (urban vs. rural), duration of unemployment (long-term vs. short-term) will be conducted to further clarify which sub-groups were reached by the programme, whether outcomes varied across sub-groups and why. This process will elucidate the contexts and mechanisms under which ASPIRE is effective and help to understand how and why the ASPIRE model works in different contexts. This will enable stakeholders and implementers including local and national authorities, employment services, small and medium-scale enterprises (SMEs) and health service providers make decisions about which interventions to embed in existing services, and how to adapt them to different circumstances.

### Study design

#### Setting

The ASPIRE project will be implemented across the 7 sites located in the FCE region. The FCE area covers the south and east coasts of England from Cornwall to Norfolk, and the north coast of France from Finistère to Pas-de-Calais. The sites in France are located in Peronne (FR223 Somme), Abbeville (FR223 Somme), Wimereux (FR302 Pas-de-Calais) and Loos-en-Gohelle (FR302 Pas-de-Calais). The sites in the United Kingdom are located in Aylesham (UKJ44 East Kent), Medway (UKJ41 Medway), and Boscombe (UKK21Bournemouth and Poole). The implementation partners are already involved in services providing healthy lifestyle, wellbeing and employability activities and have extensive experience in working with hard-to-reach, vulnerable and socially isolated individuals and communities.

#### Participants

Participants for the study will be recruited from communities within the 7 ASPIRE implementation sites. Recruitment will be carried out at community and unemployment centres, via flyers, online posts, posters and referrals from general practitioners, weight management clinics and social prescribing. Participants are eligible if they meet the inclusion criteria. Inclusion criteria will be adults (18 years above) who are unemployed, jobseekers or living with overweight or obesity (BMI between 24 kg/m^2^ and 40 kg/m^2^) using WHO classification [32] and ability to participate in activities. A lower BMI cut-off of 24 kg/m^2^ was chosen to include individuals at risk of becoming overweight and to capture participants with the risk of developing other chronic diseases. Exclusion criteria will be as follows: terminal illness or palliative care, dementia, a severe mental health problem or learning difficulty; serious health conditions that will affect uptake of intervention activities; planned bariatric or weight loss surgery; serious psychosocial problems or behavioural problems that could hinder participation in interventions (e.g. drug addiction, serious psychiatric disorders, aggressive delinquent behaviour); pregnant or planning to become pregnant and individuals currently involved in full-time paid employment or recurrent short-term contracts. Individuals who do not meet the eligibility criteria because of serious health conditions will be referred to local general practitioners or the appropriate health service provider. Ethical approval has been obtained in line with the Bournemouth University Research Ethics Committee Code of Practice (Ethics ID: 33136). Informed consent will be obtained from participants prior to enrolment in the intervention. Participants who respond to the study invitation will receive a participant information sheet with detailed information concerning the project, the nature and objectives of the study and possible risks associated with their participation and will be required to sign a consent form before participating in activities at the ASPIRE hub. All data collected for this project will be anonymised and no identifying characteristics of respondents will appear in final manuscripts, reports and publications.

#### Sample size

Power calculations to determine sample size were carried out using G Power 3.1.9.4 software (version 3) statistical power calculator. This indicated that a minimum sample size of 871 was needed to detect a weight loss difference of 1.9 kg between baseline and follow-up with a power of 90%, at α = 0.05 and effect size of 0.11. These estimations were derived from a previous study investigating the effect of cognitive behaviour therapy lifestyle intervention on weight and other health outcomes [33]. An additional sample of 15% was added to account for unpredictable effect on statistical power of clustering cases by site. To allow for attrition and ensure sufficient power during the follow up period, an attrition rate of 30% was estimated between enrolment and follow up assessment, bringing the total sample size to 1303 participants.

### The intervention

The interventions utilised in ASPIRE will focus on health-improving lifestyle activities to achieve outcomes related to weight loss, increased employability and general improvement in health and quality of life. In order to improve acceptability and enhance participation, interventions will be tailored to meet the different needs of participants who are likely to be at different stages of their weight loss or employability journey. Interventions will be co-designed with participants to ensure ownership of the model and will be adapted by the different implementation sites to suit their capacity, resources and potential participants. The ASPIRE model will consist of three interconnected elements and a fourth element which links the different elements together. The three elements include the following: (1) being active (grow your own), (2) healthy food and nutrition (eat your own), (3) achieving personal goals and improving employability (sell your own). The fourth element will be improving self-esteem through enhancing wellbeing, community engagement and accountability. It is important to note that the ASPIRE model uses a holistic approach and therefore all the elements are linked to each other via the core outcome of increasing self-esteem and support within the community. Table 1 outlines the different elements and details of interventions under each element. Activities will be delivered by trained professionals, experienced volunteers or partner agencies with experience in nutrition, health, wellbeing and employability.

**Table 1:** Overview of the core elements of the ASPIRE model and intervention activities

ASPIRE theme	Intervention activities
Self-esteem and support within a community	• Virtual reality guided meditation • Listening points • Self-care and self-image support activities • Motivational interviewing • Sophrology • Positive experience workshops • Integration into a commitment valuation system • Understanding behaviour change workshops
Grow your own - ‘Being Active’	• Setting up and maintaining community allotment or personal vegetable patch workshops • Walking groups • Understanding of seasonal and local food cultivation workshops • Fitness and wellbeing classes: yoga, tai chi, mindfulness • Health and safety in the garden workshop • Level 1 Award in Horticultural Skills
Eat your own - ‘Nutrition’	• Diet and nutrition workshops • Cooking classes • Food shop on a budget tools and support • Cooking on a budget/cooking with leftovers resources • Sourcing cheaper healthy produce • Meal planning, reducing waste and food preservation workshops. • Cooking with home grown produce • Cooking with limited resources • Food Safety in Catering Levels 1 and 2 • Level 1 Cert in Food prep and cooking
Sell your own - ‘Achieving goals and employability’	• Soft skills recognition and development workshops • Links with local business to offer volunteering and work experience • Interview preparation: mental and physical via workshops and virtual reality • Entrepreneurship support • Workshops to define skills (interpersonal skills and know-how), strengths and weaknesses • Know how to introduce yourself (in connection with the virtual reality interviews) • Interview with a referent • Level 2 Principles of COSHH (Control of Substances, Hazardous to health) • Level 2 Principles of Manual Handling • Level 3 Emergency First Aid at Work.

### Data collection

Consistent with realist principles and methodology, a mixed-method approach will be used for data collection. This will involve collecting both quantitative and qualitative data to refine the final programme theory on what aspects of the interventions in ASPIRE work, for which participants and in what context. The quantitative assessments will be used to objectively measure the outcomes which occur as a result of the intervention and qualitative assessments will provide more insights into why the interventions were effective or failed to achieve the desired outcome. Using a one-group pre-post methodology, participants will be followed up over time and data will be collected at four time points T0 (at the beginning of ASPIRE), T1 (at 12 weeks), T2 (at 6 months) and T3 (at 9 months) to map participants’ journey through the programme. At baseline (T0), the intervention will be explained to the potential participant and the consent will be obtained from the participant. This will be followed by administration of baseline questionnaires and measurement of baseline anthropometrics. All questionnaires used for data collection will be available in both French and English to ensure that no participants are excluded on the basis of language. Evaluation materials will be piloted to ensure consistency, comprehension, clarity of questions and coherence of both English and French versions and appropriate modifications will be made. A standard operating procedure for data collection has been developed and training on the evaluation and data collection process will be provided to project coordinators at the ASPIRE hubs to ensure accuracy and adherence to international standards for assessing anthropometry.

### Demographic data

Data on socio-demographic characteristics including age, gender, level of education and duration of unemployment will be collected using questionnaires.

### Primary outcomes

#### Weight, height, body mass index and body fat percentage

Weight, height, BMI and body fat percentage will be measured using the Interactive Health Kiosk (Model Number: SLF007 – Wellbeing People, Marden, Kent, England). The weighing platform of the Health Kiosk is based on load cell technology with a maximum capacity of 180 kg. Body fat percentage will be measured using multiple frequency bioimpedance analysis (BIA) technology. Weight and body fat percentage will be measured without shoes or heavy clothing to the nearest 0.1 kg and 0.1%, respectively. Height will be measured using a stadiometer (Model number: Seca 213, Seca UK) according to standard procedure for measuring height to the nearest 0.1 cm. BMI will be calculated as weight in kilograms divided by height in metres squared.

#### Employment status

**Fig. 2:**
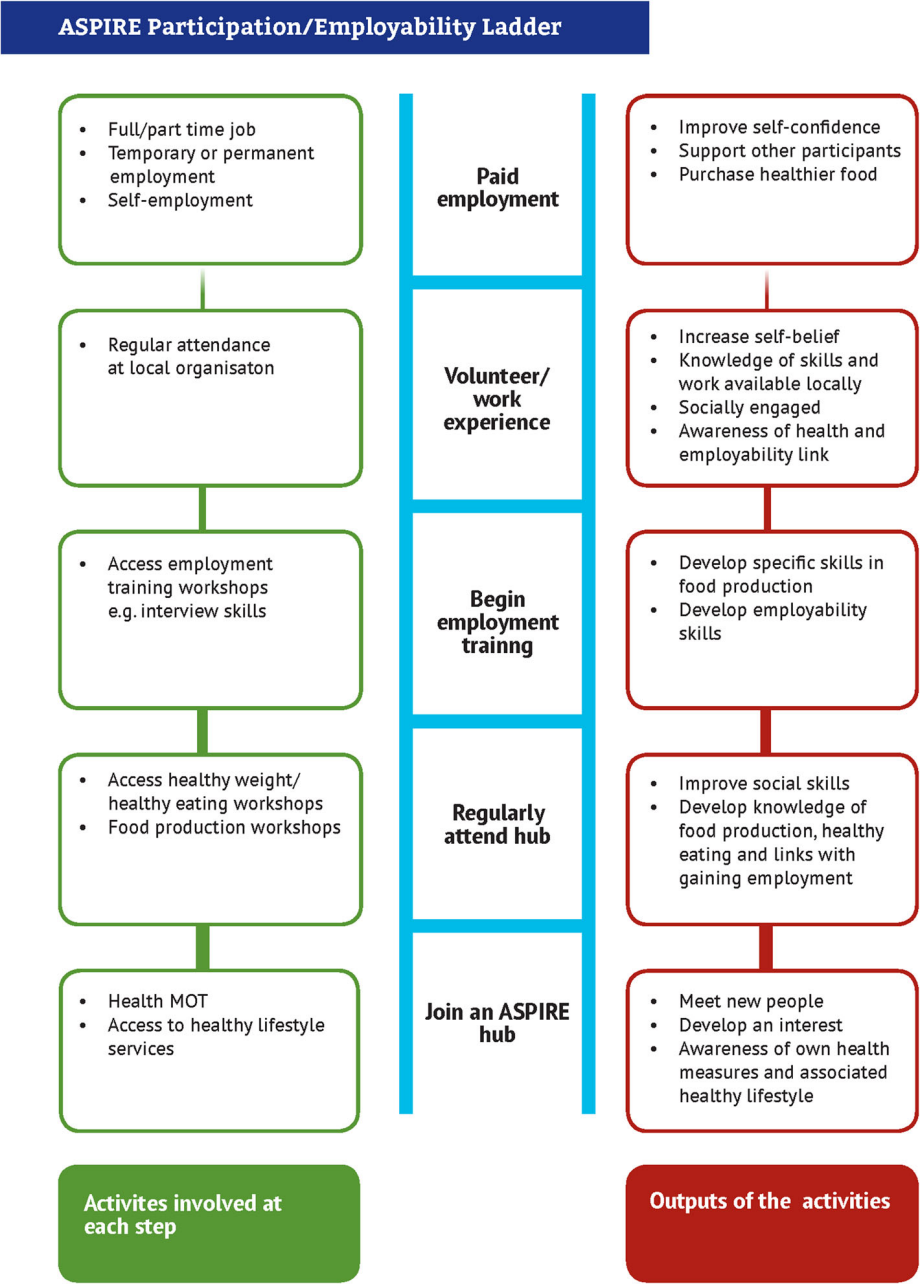
ASPIRE Participation/employability ladder. The ASPIRE participation/employability ladder describes the series of steps, activities and outputs to map participants’ progress through ASPIRE. **Abbreviations**: ASPIRE, Adding to Social capital and individual Potential In disadvantaged REgions

Employment status will be assessed by partners at local ASPIRE hubs by detailing when a participant obtains remunerated work (full-time and part-time) or subsidised work. In the analyses, employment status will be coded as a dichotomous variable (employment versus no employment) and no distinction will be made between the different types of employment. The ASPIRE participation ladder (Fig. 2) will also be used to further document participants’ status on the ladder when they join the intervention and throughout their journey to map out their rise on each stage of the ladder. The ladder consists of a series of 5 steps linked to activities and outputs to map participants’ progress through ASPIRE. The steps are as follows: ‘Join an ASPIRE hub’, ‘Regularly attend hub’, ‘Begin employment training’, ‘Volunteer/work experience’ and ‘Paid employment’.

### Secondary outcomes

#### Dietary intake and diet quality

A validated semi-quantitative food frequency questionnaire (FFQ), which includes 183 food items and a section with open questions will be used to collect data on dietary intake and diet quality. This instrument is an adapted version of the Metacardis [34] and EPIC FFQs [35, 36] to reflect the general diet in France and England and is suitable for use in both countries. Using standard portion sizes, the FFQ measures an individual’s habitual food and nutrient intake during the past year. Validation studies have shown the instrument to accurately estimate dietary intake with significant correlations with biomarkers and other dietary assessment methods.

#### Self-efficacy

This will be assessed using The General Self-Efficacy Scale (GSE) [37]. This is a 10-item self-administered scale which assesses a general sense of perceived self-efficacy with the aim of predicting how individuals cope with daily challenges and adaptation after experiencing all kinds of stressful life events. Responses are based on a 4-point scale and the sum up of responses to all 10 items yield a final composite score ranging from 10 to 40 with a higher score indicating more self-efficacy. The scale has been validated for use in general adult populations, and in samples from 23 different countries, Cronbach’s alpha ranged from 0.76 to 0.90 indicating high reliability. The GSE has been shown to positively correlate with emotion, optimism, work satisfaction. Negative coefficients were found for depression, stress, health complaints, burnout, and anxiety [38].

#### Health-related quality of life

The five-level EuroQol EQ-5D-5L questionnaire [39] will be used to assess this outcome. This instrument consists of a short descriptive system questionnaire and a visual analogue scale (EQ VAS) and provides a simple descriptive profile of a respondent’s health state. The instrument is one of the most widely used globally for measuring health status and has been proven to be valid, reliable and sensitive in varied populations [39–41].

#### Physical activity

Physical activity will be measured using self-reported assessments of physical activity on a 2-item questionnaire which reports participants’ engagement in light, moderate or vigorous physical activity over the previous week as well as the duration and frequency of the activity.

#### Health, wellbeing and activity

Self-reported health status will be assessed using 5-items on the health, wellbeing and activity questionnaire consisting of questions asking participants to rate their physical health over the past 30 days.

#### Workability

This will be assessed using 4-items adapted from the work ability and functional capacity self-assessment questionnaire [42].

### Qualitative data

Qualitative data will be obtained from participants using Photovoice. Photovoice is a community-based participatory research (CBPR) technique whereby participants identify, represent, and enhance their community through photographs and narratives [43]. As a methodology, photovoice has been used extensively in research to explore wellbeing and enabled participants to identify activities and places that enhanced their wellbeing [44]. It has also been used in studies on unemployment and food insecurity [45], and as a useful tool to engage with disadvantaged groups [46]. The technique has been shown to act as a catalyst, bringing impacts ranging from an increased sense of accomplishment to a deeper understanding of the reality of participants’ daily lives [46]. Using participatory visual methods will enable the participants to be creators of their own stories. Participants will be able to exercise control over the presentation of themselves, their wellbeing and their employability aspirations through the process, and using visual images such as photographs can empower participants to recognise their autonomy [47]. Within the ASPIRE project, a subset of participants purposively sampled will be encouraged to take up to 6 images of what the project means to their wellbeing and/or employability over 7 consecutive days and also complete a logbook documenting each photograph. These photos will be further discussed in a semi-structured interview to obtain insights into participants’ experience of ASPIRE and impact of the project on their wellbeing and employability. All interviews will be recorded on a digital Dictaphone for the purpose of transcription.

### Data analysis

Statistical analysis of quantitative data will be conducted using Statistical Package for Social Sciences (SPSS) IBM statistics (version 26, SPSS UK Ltd. Chertsey, UK). The normality of continuous variables will be checked using QQ-plots and the Shapiro-Wilk test. All tests will be carried out at the 95% confidence interval and in all analyses, a threshold of *p* < 0.05 will be considered statistically significant. Chi-square tests for independence will be used for comparing categorical variables such as sex and employment status. Means and standard deviations will be computed for continuous variables including age, body weight and BMI. Paired t-tests and repeated measures analysis will be used to examine the effect of the intervention on body weight and BMI of participants over time. Correlations and regressions or non-parametric equivalents as appropriate will be used to examine the relationships between continuous variables. The contribution of demographic variables to predicting BMI and behaviour change will be assessed using multiple linear regressions. The analysis will be conducted using intention-to-treat principles and for completers only.

Qualitative data (photos and transcript form semi-quantitative interview) will be analysed using NVivo Pro 12.5 (QSR International, 2020), which permits the coding of photos as well as text. Interviews will be audio recorded with the participants’ consent and transcribed. Thematic analysis and content analyses using both deductive and inductive approaches [48] will be used to test initial theories while allowing for emergence of new themes, and will include stages of familiarisation, coding, indexing and charting, mapping and interpretation. The deductive analysis will enable to test whether data are consistent with prior assumptions and theories identified during the development of the initial programme theory. The inductive approach which is guided by specific evaluation objectives will allow research findings to emerge from the frequent, dominant, or significant themes inherent in the raw data.

## Discussion

This paper is the first to use a mixed-methods realist evaluation to investigate the effectiveness of a novel health-related model targeted at individuals who are unemployed or living with obesity or overweight. ASPIRE is an innovative multicentre health-related and skills training programme with the aim of developing a model to holistically improve wellbeing, self-efficacy, reduce overweight, obesity and increase employment. While a consistent link between overweight, obesity and unemployment has been well established, the majority of interventions are still tailored to address these as separate entities. The ASPIRE model will regard these challenges holistically and use common strategies including improving self-efficacy, health and wellbeing and skills training to reduce overweight, obesity and unemployment. Several studies have shown the beneficial impact of reemployment on health, wellbeing and obesity [49–51] as well as the reverse impact of reducing obesity on employment outcomes [52].

The evaluation will examine which aspects of the ASPIRE model are working, for whom and in what circumstances. Both quantitative and qualitative data from the evaluation will be used to elucidate the processes of engagement and participation, which are hypothesised to mediate the programme’s success. The different contexts which enhance or reduce the uptake of intervention activities will be examined to inform how to refine existing weight and employability policies and programmes to improve their effectiveness, and how to adapt them to new contexts. Furthermore, the use of the realist approach will help to identify and test the hypothesised causal mechanisms, evaluate the extent to which ASPIRE activated them, use this analysis to refine the programme theory and identify areas of strength and potential future improvement in the programme design. The finalised theory would provide a better understanding of how mechanisms and contexts combine to generate the required outcomes in ASPIRE.

Strengths of this protocol include the use of a multidisciplinary and mixed-method realist evaluation approach to provide a better understanding of the complexity of interventions to reduce overweight, obesity and unemployment. Because of the complex and multifactorial causal factors underlying obesity and unemployment, interventions seeking to address these are equally complicated and require a comprehensive understanding of intervention context, implementation, mechanisms and outcomes which can be achieved using a realist evaluation approach. The pre-post evaluation method chosen will also minimise the effect of interindividual variation on outcomes.

In conclusion, the novel findings from this evaluation will contribute to international, European, national and regional strategies and policies to address the current challenges of obesity, overweight and unemployment. Additionally, experiences from the implementation of ASPIRE will be embedded in existing services to enhance the quality and effectiveness of interventions that individuals who are overweight, obese or unemployed can receive. Furthermore, participants and their families will benefit immensely through the maintenance of a healthy lifestyle and the model can be adapted by any community looking to make healthy lifestyle changes.

## Supplementary Information

**Additional file 1: Supplementary table 1**: Items from the World Health Organization Trial Registration Data Set.

## Data Availability

Data sharing is not applicable to this article as no datasets were generated or analysed during the current study.

## Abbreviations

BMI: Body mass index
CMO: Context-mechanism-outcome
CBPR: Community-based participatory research
FCE: France Chanel England
FFQ: Food frequency questionnaire
GSE: General self-efficacy
PA: Physical activity
RE: Realist evaluation
WHO: World Health Organisation
