# Supplementary material for: Evaluation of a health-related intervention to reduce overweight, obesity and increase employment in France and the United Kingdom: a mixed-methods realist evaluation protocol
Source: BMC Public Health. 2021 Mar 24;21:582. doi: 10.1186/s12889-021-10523-3 (PMC7987742; doi:10.1186/s12889-021-10523-3)
Supplement: Supplementary file 1 — Additional file 1: Supplementary table 1: Items from the World Health Organization Trial Registration Data Set. [file 12889_2021_10523_MOESM1_ESM.docx]

**Supplementary table 1**: Items from the World Health Organization Trial Registration Data Set

| **Data category** | **Information** |
| --- | --- |
| Primary registry and trial identifying number |  |
| Date of registration in primary registry |  |
| Secondary identifying numbers |  |
| Source(s) of monetary or material support | EU Interreg European Regional Development Fund |
| Primary sponsor | Bournemouth university |
| Contact for public queries | Prof Jane Murphy (jmurphy@bournemouth.ac.uk) |
| Contact for scientific queries | Prof Jane Murphy (jmurphy@bournemouth.ac.uk) |
| Public title | Adding to Social capital and individual Potential In disadvantaged REgions |
| Scientific title | Adding to Social capital and individual Potential In disadvantaged REgions |
| Countries of recruitment | United Kingdom, France |
| Health condition(s) or problem(s) studied | Overweight, obesity, unemployment |
| Intervention(s) | Healthy lifestyle activities |
| Key inclusion and exclusion criteria | *Inclusion criteria:*  •Individuals who are overweight or obese or unemployed or jobseeker  •Ability to attend activities at ASPIRE hub in the different implementation sites  •Adults 18 years above  *Exclusion criteria:*  •Terminal illness or palliative care, dementia, a severe mental health problem or learning difficulty.  •Planned bariatric or weight loss surgery.  •Serious psychosocial problems or behavioural problems that could hinder participation in interventions (e.g. drug addiction, serious psychiatric disorders, aggressive delinquent behaviour).  •Pregnant or planning to become pregnant.  •Individuals currently involved in full-time paid employment or recurrent (continuous) short-term contracts. |
| Study type | Interventional |
| Date of first enrolment | September 2020 |
| Target sample size | 1303 |
| Recruitment status | Recruiting |
| Primary outcome(s) | Decrease in body weight and body mass index (BMI) and increase in employment |
| Key secondary outcomes | Rise on the ASPIRE employability ladder, improvement in diet quality, physical activity, health-related quality of life, self-efficacy and health and wellbeing. |
